# Exploration and demonstration of explainable machine learning models in prosthetic rehabilitation-based gait analysis

**DOI:** 10.1371/journal.pone.0300447

**Published:** 2024-04-02

**Authors:** Mohammad Pourmahmood Aghababa, Jan Andrysek

**Affiliations:** 1 Institute of Biomedical Engineering, University of Toronto, Toronto, Ontario, Canada; 2 Bloorview Research Institute, Holland Bloorview Kids Rehabilitation Hospital, Toronto, Ontario, Canada; Air University, PAKISTAN

## Abstract

Quantitative gait analysis is important for understanding the non-typical walking patterns associated with mobility impairments. Conventional linear statistical methods and machine learning (ML) models are commonly used to assess gait performance and related changes in the gait parameters. Nonetheless, explainable machine learning provides an alternative technique for distinguishing the significant and influential gait changes stemming from a given intervention. The goal of this work was to demonstrate the use of explainable ML models in gait analysis for prosthetic rehabilitation in both population- and sample-based interpretability analyses. Models were developed to classify amputee gait with two types of prosthetic knee joints. Sagittal plane gait patterns of 21 individuals with unilateral transfemoral amputations were video-recorded and 19 spatiotemporal and kinematic gait parameters were extracted and included in the models. Four ML models—logistic regression, support vector machine, random forest, and LightGBM—were assessed and tested for accuracy and precision. The Shapley Additive exPlanations (SHAP) framework was applied to examine global and local interpretability. Random Forest yielded the highest classification accuracy (98.3%). The SHAP framework quantified the level of influence of each gait parameter in the models where knee flexion-related parameters were found the most influential factors in yielding the outcomes of the models. The sample-based explainable ML provided additional insights over the population-based analyses, including an understanding of the effect of the knee type on the walking style of a specific sample, and whether or not it agreed with global interpretations. It was concluded that explainable ML models can be powerful tools for the assessment of gait-related clinical interventions, revealing important parameters that may be overlooked using conventional statistical methods.

## Introduction

Clinicians commonly use gait indicators or parameters—such as the step length, stride velocity, and joint angles—to diagnose gait issues and establish a suitable course of rehabilitation [1]. Gait metrics also play an important role in research, facilitating the assessment of interventions that are most effective in improving gait [2, 3]. Gait parameters are typically obtained via instrumented gait analysis techniques, such as optical cameras calibrated to track the body in three-dimensional space or wearable motion sensors (i.e. inertial measurement units). These technologies allow for large sets of gait parameters (spatiotemporal and kinematic) to be accurately measured and analyzed.

The two main techniques for analyzing interventional differences in gait data are traditional statistical methods and machine learning (ML) [4]. Statistical methods, such as analyses of variance, are most commonly used in clinical research as they are easily understood and applied, in addition to providing measures of association with the dependent variable. However, statistical methods rely on strong assumptions, such as the type of residual distribution, linear dependency of the variables with the dependent variable, and independency of the predictors [4–6]. Further, these methods are also not ideal for applications with small sample sizes and large sets of variables, as is common in rehabilitation research using quantitative gait analysis methods [4, 7]. Traditional statistical methods can result in underpowered analyses prone to type I errors or the potential discovery of gait changes that may not be important [8]. A common way of dealing with this issue is hypotheses-based gait parameter reduction, where the researcher selects a sub-group of parameters based on the anticipated changes in gait (associated with the investigated intervention); however, this can result in the loss of important parameters and insights into unforeseen performance effects [9].

Recently, there has been a growing interest in the application of ML models for gait analysis methods, as they can address some of the shortcomings of traditional statistical methods and provide flexibility, scalability, and independence from inferred assumptions [10–14]. ML methods can also be applied to different data types (e.g. images, text, and tabular data), and the outcomes can be merged into predictions for diagnosis, prognosis, and possible treatments [15, 16]. ML approaches, such as principle component analysis, can reduce the volume of data and improve visualization, which can help determine similarities and differences between samples [17]. Other ML methods, including support vector machine (SVM) algorithms, were successfully used for the automatic classification of prosthetic components, despite relatively small sample sizes [10]. In [11], a k-nearest neighbour method was successfully used to classify the abnormal gait patterns caused by knee osteoarthritis and Parkinson’s disease. In [12], four different ML models, including partial least square-discriminant analysis, SVM, random forest (RF), and artificial neural networks, were used to extract and use informative features for classifying gait characteristics according to certain subject characteristics. Hence, it is clear that ML models can leverage large amounts of data in an automated manner to ascertain the nonlinear and complex interactions between predictors and dependent variables. However, a challenge with ML techniques is their black-box nature. It can be difficult to understand the complex underlying mechanisms of ML models and the results that they yield [18]. This has led to significant research interest in explainable ML models for gait analysis [19].

Few studies have investigated explainable ML for clinical gait classification. For example, the work in [20] used explainable ML with a gait dataset of ground reaction force measurements to successfully recognize significant gait parameters and quantify their contribution to the diagnosis of anterior cruciate ligament injury. Similarly, the authors of [21] used tree-based explainable ML models to identify the most important factors affecting the gait speed of elderly people. In [22], the feature importance characteristics of two tree-based ML methods (XGBoost and RF) were used to determine the significance of statistical gait parameters on osteopenia and sarcopenia, which could ultimately lead to better management measures for these issues in the elderly. A fuzzy logic-based feature selection for knee osteoarthritis was developed in [23] and Shapley Additive exPlanations (SHAP) [24] was used to determine the importance of the chosen features and the rationale behind the decision-making process of the model. In [25], local interpretable model-agnostic explanation (LIME) was employed for identifying the most important gait parameters used in foot disorders and surgery planning. Although LIME successfully recognized the significance of the gait parameters for several ML models in [25], unlike SHAP, its results could not be easily generalized for a wider population.

Permutation feature importance (PFI) [26], LIME, and SHAP are three different techniques in the realm of explainable machine learning that have been used for interpreting and understanding the outputs of ML models. PFI works by permuting (randomly shuffling) the values of a single feature and measuring the change in model performance. Then, the feature importance is determined by the decrease in model performance when the feature’s values are randomly permuted. A larger drop in performance indicates a more important feature. PFI provides only a global interpretation of feature importance, i.e., how each feature contributes to the overall model performance. On the other hand, LIME focuses on explaining the predictions of an ML model by approximating the model’s decision boundary locally using a simpler, interpretable model (e.g., linear model). LIME provides local explanations for a specific instance or a small set of instances. This method creates perturbed versions of the input data, observes the corresponding predictions, and fits an interpretable model to approximate the complex model locally. LIME only offers instance-specific explanations and may not capture global patterns in the data. However, SHAP values are based on cooperative game theory and Shapley values. SHAP methodology assigns a value to each feature, representing its contribution to the prediction for a particular instance. SHAP values provide a way to allocate the prediction value among the features. SHAP analysis can be used for both local and global interpretability. In conclusion, PFI offers a global view of feature importance based on model performance, LIME provides local, instance-specific explanations using simplified models, and SHAP values offer a comprehensive approach to understanding feature contributions at both the local and global levels.

In the domain of explainable ML, local and global interpretation refer to different scopes or levels at which the interpretation or explanation of a model’s predictions is conducted. Local interpretation aims to explain the predictions of a model for a specific sample in the dataset. For local interpretation with SHAP, sample SHAP values are computed for each feature for a given sample. In the case of clinical research, this may be the observations of a particular patient. These values represent the contribution of each feature to the difference between the model’s prediction for that instance and the expected model prediction which usually is the average prediction for the dataset. Local interpretation is useful for understanding why a particular prediction was made for a specific instance. It provides insights into the factors influencing the model’s output at a sample level. On the other hand, global interpretation looks at the overall behavior of the model across the entire dataset. For global interpretation with SHAP, aggregate statistics such as feature importance or summary plots are used to understand the general patterns and trends in feature contributions across all instances in the dataset (overall sampled population). Global interpretation is valuable for gaining insights into the overall behavior of the model, identifying important features across the entire dataset, and understanding which features consistently contribute to predictions. Unlike the local interpretation which is useful for explaining a specific prediction or providing instance-specific justifications, global interpretation is employed when understanding the model’s behavior at a broader level is required to identify important features across the dataset and make generalizations about feature contributions. Overall, local interpretation in SHAP analysis involves explaining sample predictions, while global interpretation focuses on understanding the overall behavior of the model across the entire dataset. Both perspectives contribute to a comprehensive understanding of a model’s predictions and can be valuable in different contexts, depending on the specific goals of the analysis.

Owing to its ability to analyze both population and sample-based outcomes, along with its high performance in terms of explaining ML models, SHAP is utilized to analyze gait changes related to physical rehabilitation in this study. The objective is to explore and demonstrate the use of ML-based classifiers to differentiate between two types of prosthetic interventions using gait parameters as model inputs, and to apply explainable ML techniques to interpret ML models and identify the most influential gait parameters. A complementary objective of this study is to develop both population- and sample-based explainable models for clinical prosthetic gait analysis using explainable ML.

## Methods

The dataset comprised gait patterns for two prosthetic knee joints captured using sagittal-plane video recordings of 21 individuals with transfemoral amputations. The participants were recruited between Dec 2016 and Sept 2017. The study was approved by the National Ethics Committee for Health Research, Cambodia (002NECHR), and Bloorview Research Institute Ethics Board (REB16-686). Written consent was obtained from all participants prior to data collection. The author (JA) has access to information that could identify individual participants during or after data collection.

### Experimental protocol

In a crossover study design, participants conducted walking trials during two separate sessions with two prosthetic knee joints, including the All-Terrain knee (Legworks, Inc., Toronto, ON, Canada) and the International Committee of the Red Cross knee joint (CREquipments, Coppet, Switzerland), hereinafter referred to as the ATK and ICRC, respectively. The participants were instructed to walk along a 10-meter walkway at their normal and fast-walking speeds in both directions to capture videos of at least on full stride bilaterally. A total of 19 features were extracted from the video files using open-source software (Kinovea), including both spatiotemporal and kinematic gait parameters, as listed in Table 1. Symmetry indices (SI) were computed for the swing time, step length, and maximum knee flexion to enable the direct comparison of the intact and prosthetic limbs.

$$SI=\frac{V_{prosthetic}-v_{intact}}{0.5\left(V_{prosthetic}+v_{intact}\right)}\times 100$$ (1)

where V represents gait parameter recorded on the prosthetic/intact limb.

**Table 1:** Descriptions of the extracted gait parameters.

Gait Parameter	Description (units)
Prosthetic ROM Knee	Angular range-of-motion at prosthetic knee (deg)
Intact ROM Knee	Angular range-of-motion at intact knee (deg)
Prosthetic Max Knee Flex	Maximum knee flexion at prosthetic knee—heel rise (deg)
Intact Max Knee Flex	Maximum knee flexion at intact knee—heel rise (deg)
Prosthetic Max Hip Flex	Maximum hip flexion at prosthetic hip (deg)
Intact Max Hip Flex	Maximum hip flexion at intact hip (deg)
Stride Length	Distance between successive ipsilateral foot contacts (m)
Stride Velocity	Velocity tracked via ankle (m/sec)
Stride Velocity CI	Velocity tracked via iliac crest (IC) (m/sec)
Cadence	Walking rate (steps/minute)
Double Support Time	Time that both feet are contacting the ground (sec)
Prosthetic Step Length	Distance between prosthetic and intact foot contacts (m)
Intact Step Length	Distance between intact and prosthetic foot contacts (m)
Prosthetic Swing Time	Time between foot-off and foot-strike on prosthetic side (sec)
Intact Swing Time	Time between foot-off and foot-strike on intact side (sec)
ROM Y Displacement	Vertical displacement of the iliac crest markers (m)
Swing Time SI	Symmetry index of swing time based (unitless)–see Eq 1
Step Length SI	Symmetry index of step length (unitless)–see Eq 1
Knee Flex SI	Symmetry index of maximum knee flexion (1)–see Eq 1

### Analytical methods

Logistic/Logit regression (LR) [27] is a parametric classification algorithm used to determine the likelihood of a particular data input belonging to a binary class. This method multiplies each feature X = (x_0_, x_1_, x_2_, …, x_n_) by a regression weight W = (w_0_, w_1_, w_2_, …, w_n_) and calculates their summation. Then, it adds a bias term ‘b’ to be fed to a sigmoid function (i.e. S-shaped function), given in Eq (2). A gradient-based numerical algorithm is usually used to find the (sub)optimal coefficients.


$$\sigma \left(t\right)=\frac{1}{1+exp(-t)}$$ (2)

SVM [2] is a simple but powerful ML modelling approach for both linear (and nonlinear) classification and regression purposes. In the case of linear separable binary classification problems, a hyperplane separates two different classes. For a given dataset {(x_1_, y_1_), (x_2_, y_2_),…, (x_N_, y_N_)} with N instances, SVM establishes a hyperplane by finding a weight vector W and bias b. The goal is to minimize the term ||w||^2^, as follows.


$$Minimize:\frac{1}{2}W^{T}W,\;S.t.:y_{i}\left(W^{T}x_{i}+b\right)\geq 1,\;i=1,\;2,\;\ldots ,\;N$$ (3)

RF [28] implements a supervised learning strategy for both classification and regression problems. In an RF algorithm, a forest of decision trees is created and combined to construct a model with predictions. The average (or median) of the outcome and majority vote strategy are used from different trees to forecast or classify data. RF models are able to evaluate the relative importance of applied features by investigating the average amount of impurity reduction in tree nodes that deal with those features.

LightGBM [28] is a newer ML model, which adopts a gradient-boosting decision tree algorithm for higher-speed execution, lower memory usage, and higher precision than conventional tree-based methods. The LightGBM algorithm uses a histogram to compute the gradients. Similar to RF, LightGBM has the ability to assess the importance of features.

SHAP [24] is a library that helps compute Shapley values and visualize these values in understandable and comparable ways. It is used for explaining the outputs of ML models by calculating and examining the contribution of input variables. SHAP decomposes the output into a sum of contributions from each variable feature of an input and presents the predictions as the sum of SHAP values added to a fixed base value. From an analytical standpoint, SHAP attempts to explain sample predictions (i.e. outputs) based on the game theory optimal Shapley values [24]. In general, SHAP analysis can determine the contribution and importance of each feature in the predictions of ML models for both the entire population as well as each sample.

Various preprocessing methods were carried out to prepare the raw data for the ML models. To determine and exclude outliers stemming from the video recording process and/or gait parameter extraction from the videos, the IQR algorithm was used [29]. Accordingly, first lower and upper boundaries of quantiles were calculated via L_f_ = Q1 − 1.5 × IQR and U_f_ = Q3 + 1.5 × IQR, where IQR represents interquartile range (i.e. upper quartile—lower quartile). Then, all samples outside of the range [L_f_, U_f_] were identified and removed as outliers. For excluding redundant gait parameters, Pearson’s correlation analysis was used. The prosthetic ROM knee, intact ROM knee, and stride velocity IC were highly correlated (R^2^ > 0.9) and, therefore, removed from the dataset. As an essential step in building ML models, the data was shuffled to ensure that the samples were identically and independently distributed. More specifically, this shuffling could assist the gradient-based optimization algorithms in finding accurate optimal weights for the ML models. Since the gait parameters had different units and ranges of values, to avoid the dominance of some gait parameters over others, the Min-Max-Scaler function was used to map the range of the data into [0, 1]. This preprocessing technique removes the scale of the data and increases the stability of the ML models and decreases the odds of approaching nonoptimal weights of the ML models [27]. Since the data were complete, no missing data imputation was carried out. Also, as there was no categorical feature in the data, no encoding method was employed for converting categorical features into numerical values. Finally, 75% of the data was randomly selected as the training dataset, and the remainder was left for the test set.

The four classifiers were assessed against three performance indicators: the accuracy, receiver operating characteristic curve area under the curve (ROC-AUC), and F_1_ score. The accuracy measures how many predictions, including both positive and negative cases, were correctly classified. The F_1_ score computes the harmonic mean between precision and recall. The Precision is defined as the proportion of positive predictions that was actually correct. The Recall represents the proportion of actual positives that was predicted correctly. The ROC plots true positive rate against false positive rate and, therefore, the ROC-AUC score measures the area underneath the ROC. The following equations were used to calculate the metrics.

$$Accuracy=\frac{TP+TN}{TP+TN+FP+FN}$$

$$Precision=\frac{TP}{TP+FP}$$

$$Recall=\frac{TP}{TP+FN}$$

$$F_{1}=\frac{2*Precision*Recall}{Precision+Recall}=\frac{TP}{TP+0.5(FP+FN)}$$ (4)

where TP, TN, FP, and FN stand for true positive, true negative, false positive, and false negative samples, respectively.

Finally, to determine the best hyperparameters, a K-fold grid search cross-validation algorithm was implemented. First the training dataset was split into five folds. Then, the first fold was used as the test dataset and the rest of the folds were applied for training the ML models. This was repeated with the second fold as the testing dataset and the remainder for training and so on. In each iteration, an exhaustive search over specified hyperparameter values for each classifier was performed (i.e. all the possible combinations of the hyperparameter values were considered and applied). The scores were computed to find the best hyperparameters as given in Table 2.

**Table 2:** Hyperparameters obtained for the classifiers.

Model	Hyperparameter space	Best hyperparameters
LR	regularization: [‘none’, ‘l1’, ‘l2’, ‘elasticnet’] C: [100, 10, 1.0, 0.1, 0.01]	regularization: ‘l2’ C: 0.2
SVM	kernels: [‘linear’, ‘poly’, ‘rbf’, ‘sigmoid’] C: [100, 10, 1.0, 0.1, 0.001] gamma: [‘scale’, ‘auto’]	kernels: rbf C: 1.0 gamma: ‘scale’
RF	max_depth: [5, 15, 25, 35, 45], n_estimators: [10, 50, 100, 150, 200], min_samples_split = [3, 5, 8, 1010, 15], max_leaf_nodes: [10, 50, 100, 150, 200], max_features: [’auto’, ’sqrt’, ’log2’]	max_depth = 25 n_estimators = 100 min_samples_split = 5 max_leaf_nodes = 100 max_features = ’log2’
LightGBM	num_leaves: [2, 5, 10, 15] min_data_in_leaf: [2, 5, 7, 10, 15, 18] max_depth: [2, 4, 6, 8, 10] feature_fraction: [0.25, 0.5, 0.75, 1] learning_rate: [0.01, 0.05, 0.1, 0.3, 0.5, 0.9] max_bin: [10, 20, 25, 30, 35, 40] num_iterations: [5, 25, 50, 100, 150, 200]	num_leaves = 5 min_data_in_leaf = 7 max_depth = 6 feature_fraction = 1 learning_rate = 0.05 max_bin = 35 num_iterations = 150

**Remark 1**. Initially, the analysis was performed with seven models namely LR, SVM, LightGBM, RF, XGBoost, Catboost, and a dense neural network. Since the results of tree-based methods XGBoost and Catboost were similar to the results of LightGBM, in order to have a concise report without losing informative results, their results were removed from the analysis. Also, the results of the developed dense neural network were not as good as the results of the other methods which could be due to the low number of dataset’s samples. So, the neural network model was not included in the report either. It is noted that the experiments were conducted with diverse hyperparameters for all the models and experiments were repeated for various times to have a benchmark and solid analysis.

**Remark 2**. To examine the type of the relationship between gait parameters and the two knees, both (semi)linear and nonlinear ML models were employed. The first option was to use a logistic regression (considering that its outputs are passed through a sigmoid function). Then the complexity of the model was increased by implementing an SVM model with both linear and nonlinear kernels. To investigate further the relationship between gait parameters and the knees, the third model was chosen as an RF where it has been known to detect the nonlinear relationships between dependant and independent variables. The gradient-boosting decision tree LightGBM model was also used as a potential alternative to RF where it has been known to be fast and accurate. However, tuning the LightGBM was more complicated compared to the RF model and it tended to overfit in the experiments. After running the models for at least 15 times, the mean of the results was chosen as the statistical indicator for the performance evaluation of the models.

## Results

### Classification results

**Fig 1:**
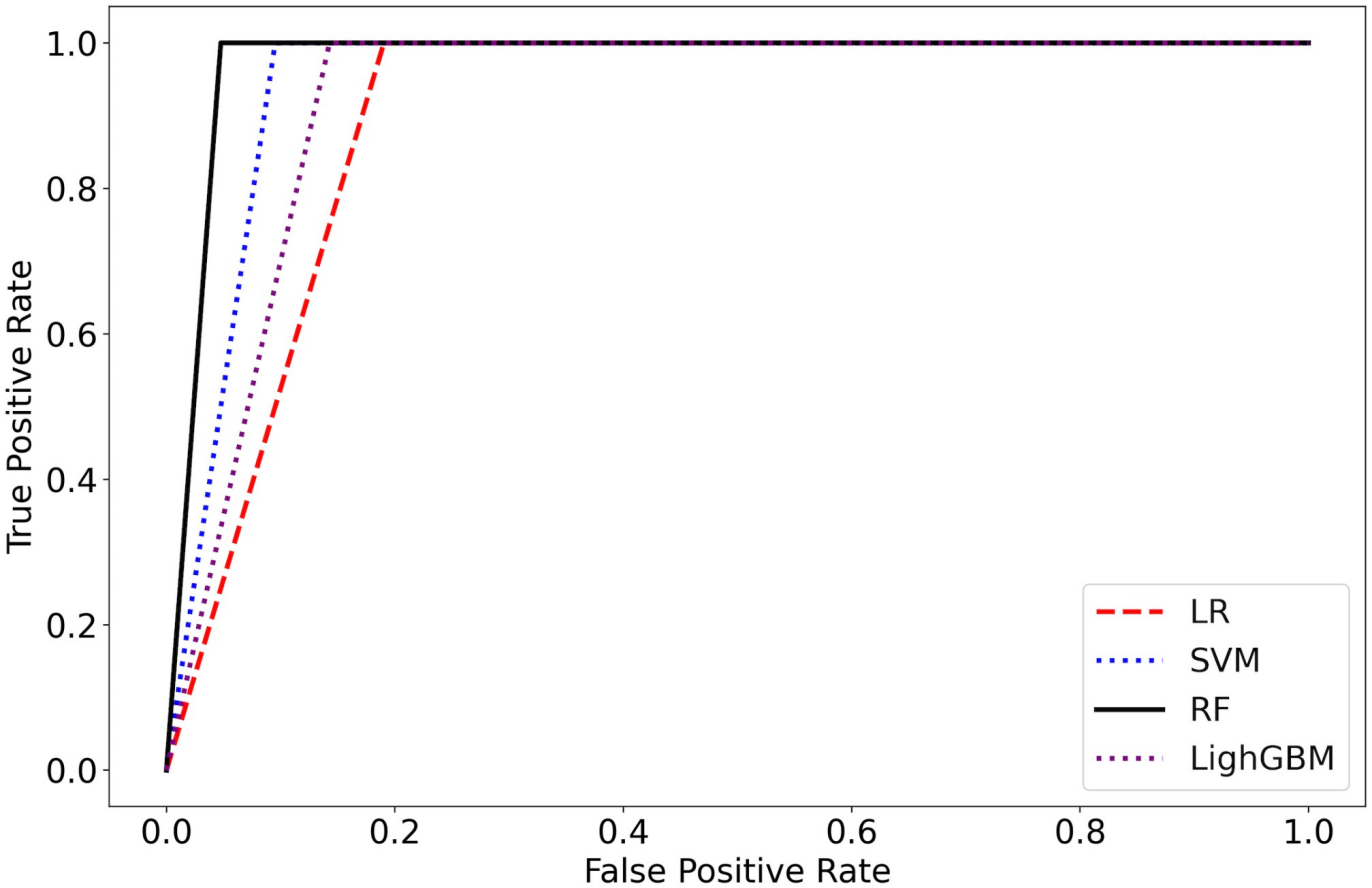
ROC curves of the adopted ML models.

Since randomness usually exists when generating different initial parameters for ML models (e.g. initialization of weights, shuffling the data, etc.), the developed models were run 15 times to find the best results. All ML models performed well (>90%, except for ROC-AUC of LR and SVM) when classifying the knee joints; the RF model performed the best as shown in Table 3. Also, the corresponding ROC curves were illustrated in Fig 1.

**Table 3:** Performance of classifiers on test data.

Model	Accuracy (%)	F_1_ score (%)	ROC-AUC (%)	Precision	Recall
LR	91.0	92.5	84.5	90.3	93.3
SVM	91.0	92.6	89.1	90.3	97.2
RF	**98.3**	**98.7**	**97.5**	**97.6**	**98.5**
LightGBM	93.3	95.4	90.0	95.4	96.1

### Feature importance for tree-based models

**Fig 2:**
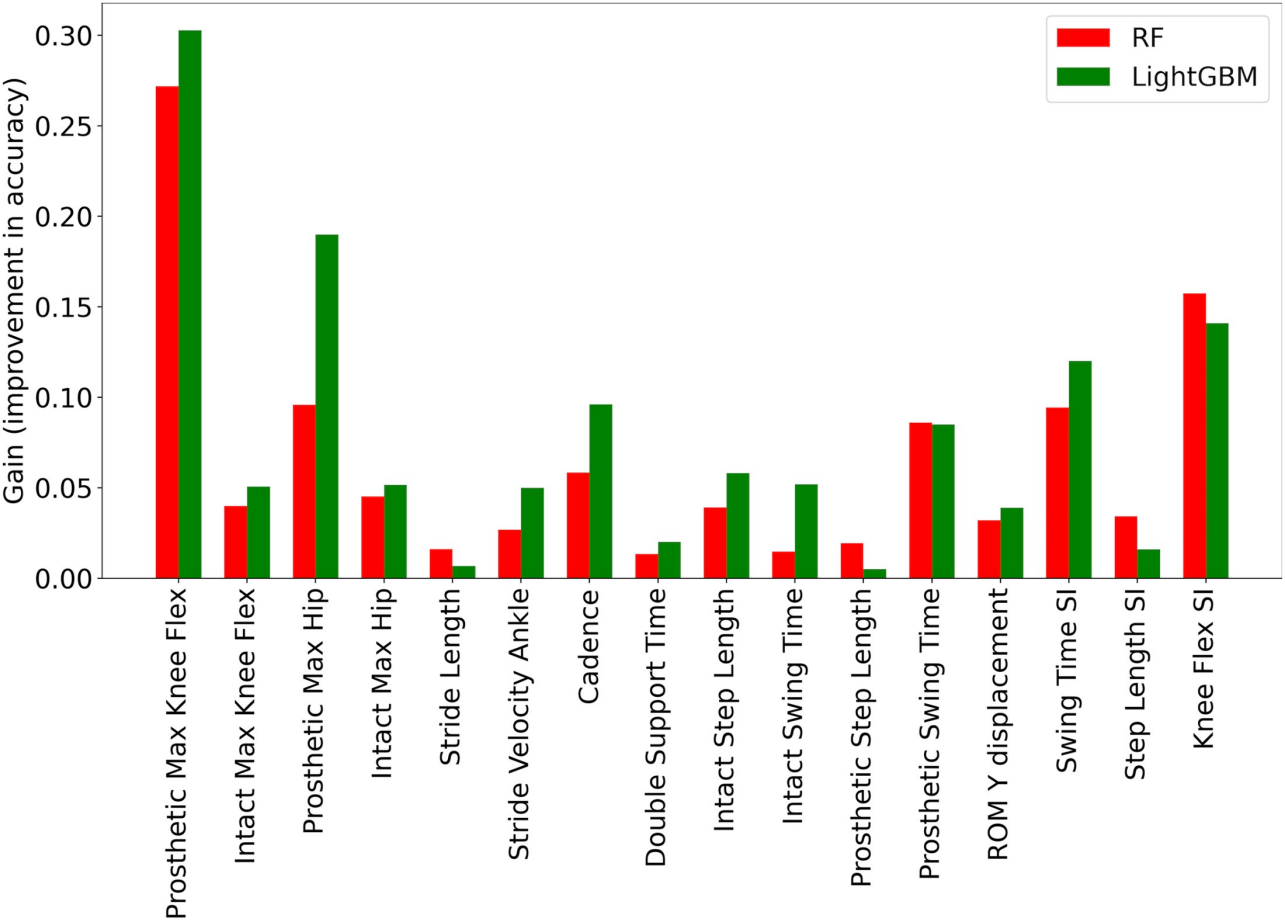
Feature importance for the RF and LightGBM models.

Tree-based algorithms, such as RF, XGBoost, CatBoost, and LightGBM, can provide an analysis of the importance of features. This can reveal how influential each feature (e.g. gait parameter) is in determining the output of the model. The feature importance diagrams of the developed RF and LightGBM models are shown in Fig 2(a) and 2(b), respectively. It can be seen that the two most important gait parameters were prosthetic max knee flexion and knee flexion SI, implying that the two parameters were most affected by the type of prosthetic intervention. The least influential gait parameters for both models were identified as double support time, stride length, and prosthetic step length. These gait parameters did not change significantly due to the given intervention.

### SHAP global interpretability: Explaining population predictions

**Fig 3:**
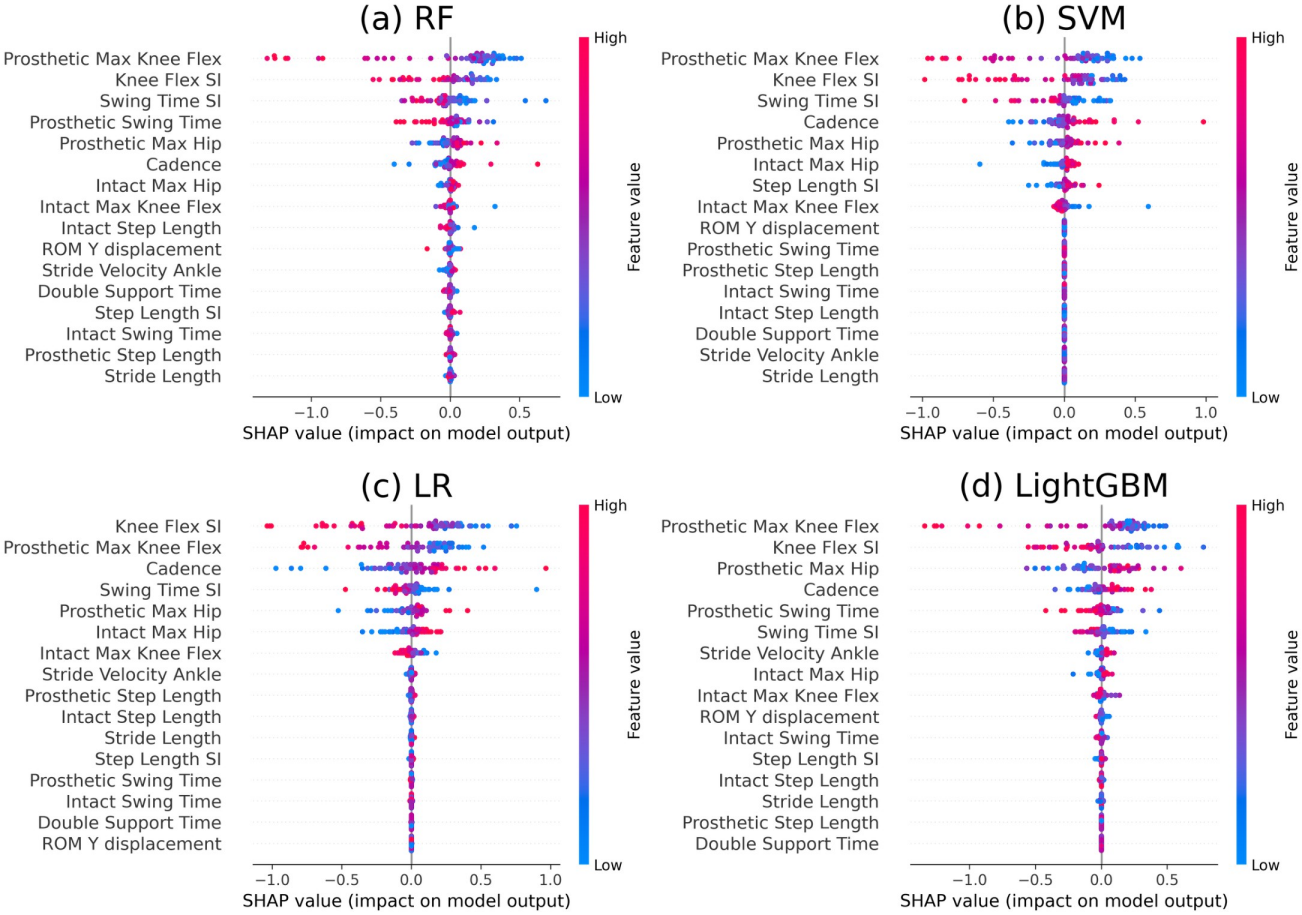
Global interpretability for (a) RF, (b) SVM, (c) LR, and (d) LightGBM. The blue dots and red dots on the right side of the vertical line indicate low and high values, respectively, of corresponding gait parameters that have an influence on ATK. Similarly, the dots on the left side of the vertical line are for ICRC. Features at the top are of the most importance. For example, for Prosthetic Flex SI, the ATK has lower values (i.e. less prosthetic knee flexion) compared to ICRC. The changes in the gait parameters are due to the ATK swing-phase control mechanism. The ATK mechanism better controls (compared to ICRC) the kinematics at the knee joint during gait to increase symmetry between the prosthetic and non-prosthetic sides, specifically related to swing times, step lengths as well as heel-rise (swing-phase knee flexion excursions) across different walking speeds. For a more detailed explanation please refer to [3].

SHAP global interpretability yields the rank of the influence of each gait parameter [24]. It also determines the direction of influence. Global SHAP values for the RF, SVM, LR, and LightGBM algorithms are shown in Fig 3(a)–3(d), respectively. In these plots, by default, the features were ordered using the mean absolute value of the SHAP values for each feature. In all four models, the prosthetic max knee flex and knee flex SI were identified as the most influential/top ranked gait parameters. ATK led to lower values of prosthetic max knee, knee flex SI, swing time SI, prosthetic swing time, and intact max knee flex compared to ICRC. On the other hand, cadence, prosthetic max hip, and stride velocity ankle parameters were higher for ATK.

**Remark 3**. In order to find the most important features of the models by another method, the recursive feature elimination (RFE) algorithm was adopted. RFE is a feature selection technique used to iteratively remove less important features from a model based on their importance ranking. RFE starts by training the model on the entire set of features. Then, it ranks the features based on their importance scores. These scores indicate the contribution of each feature to the model’s performance. Afterward, the least important feature (according to the ranking) is removed from the feature set. The model is then retrained using the remaining features. These steps are repeated iteratively until a specified number of features is reached or until the model’s performance (e.g., accuracy) stabilizes or starts to degrade. The final set of selected features, obtained after the recursive elimination process, represents the subset that the algorithm determined to be the most relevant for the given ML task. In this work, the RFE was implemented for all four models assuming that the final number of features to select was six. The results of the RFE were shown in Table 4. It is noted that RFE did not provide the rank and amount of contribution of the selected features. Also, the results of the RFE method could change based on the number of features to be selected.

**Table 4:** RFE results for the ML models.

Model	Most important features selected by RFE
LR	Stride Length, Stride Velocity Ankle, Cadence, Intact Step Length, Prosthetic Step Length, Prosthetic Swing Time
SVM	Prosthetic Max Knee Flex, Intact Max Knee Flex, Intact Swing Time, Prosthetic Step Length, Prosthetic Swing Time, Knee Flex SI
RF	Prosthetic Max Knee Flex, Prosthetic Max Hip, Cadence, Prosthetic Swing Time, Swing Time SI, Knee Flex SI
LightGBM	Prosthetic Max Knee Flex, Intact Max Knee Flex, Prosthetic Max Hip, Cadence, Prosthetic Swing Time, Knee Flex SI

### Independence plots: Explaining single feature impact

SHAP dependence plots are used for exploring the impact of a single feature on the model’s predictions while accounting for the effects of other features. In this paper, they provide a clear visual representation of how the output of the developed ML model changes with variations in a specific gait parameter while considering the effects of other features. To depict the SHAP dependence plots, first SHAP values for each instance in the dataset are computed to represent the contribution of each feature to the difference between the model’s prediction for a specific instance and the expected model prediction. Then a target feature for the dependence analysis is chosen. Subsequently, a scatter plot with the x-axis representing the values of the chosen gait parameter and the y-axis representing the corresponding SHAP values for that parameter is depicted. Each point on the scatter plot corresponds to an instance in the dataset. Finally, analyzing the shape and trend of the dependence plot reveals the amount of the changes in the chosen feature’s values associated with changes in the model’s predictions.

**Fig 4:**
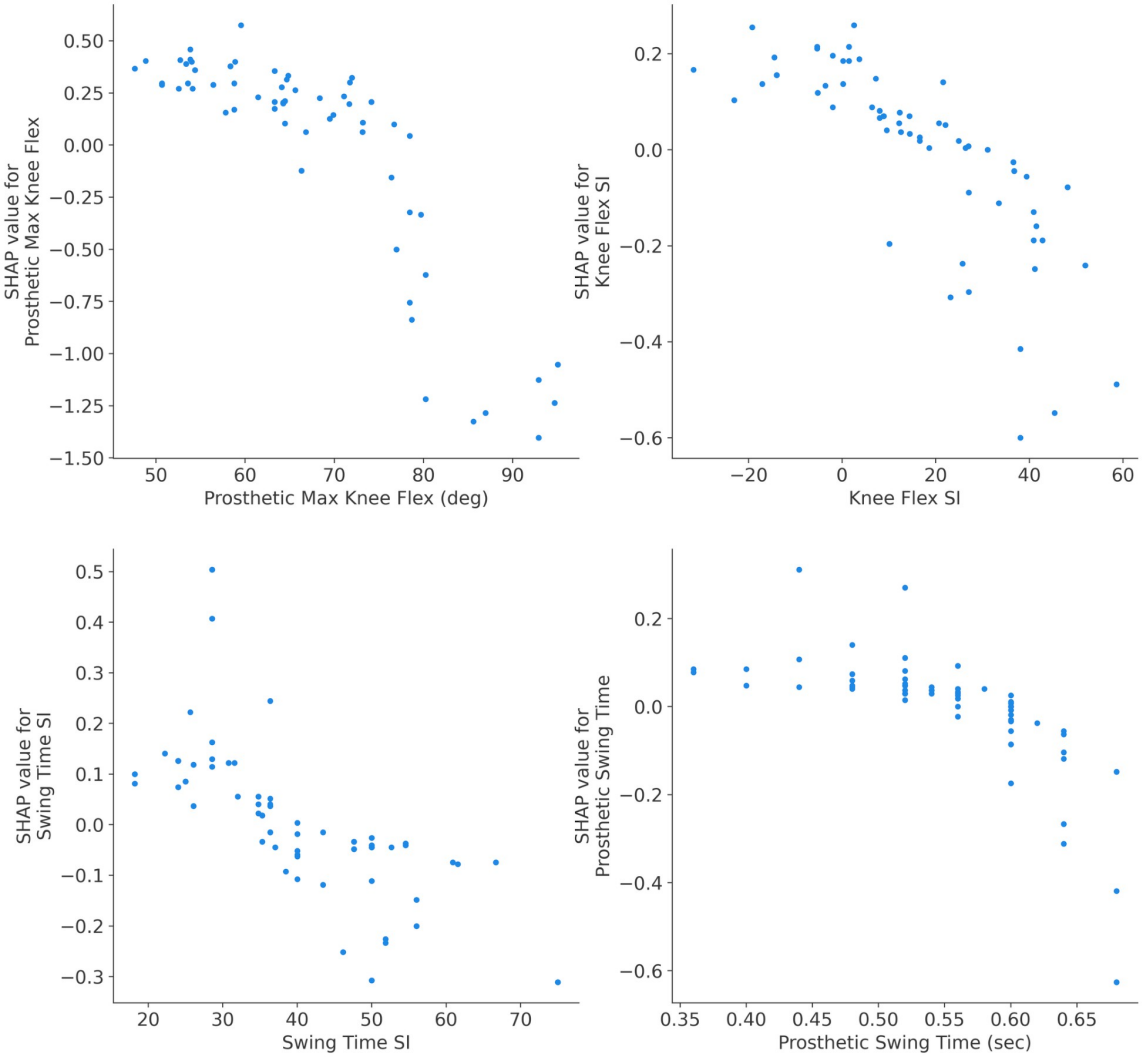
SHAP dependence plots for four most influential decreasing gait parameters via RF model. For the low values of prosthetic max knee flex (up to 73 degrees), the likelihood of wearing an ATK was high. After passing 73 degrees, the probability of wearing an ATK started to decline meaning that the ICRC knee was likely worn. A similar pattern was observed for knee flex SI, swing time SI, and prosthetic swing time with the critical points of roughly 7, 33, and 0.52 sec, respectively.

**Fig 5:**
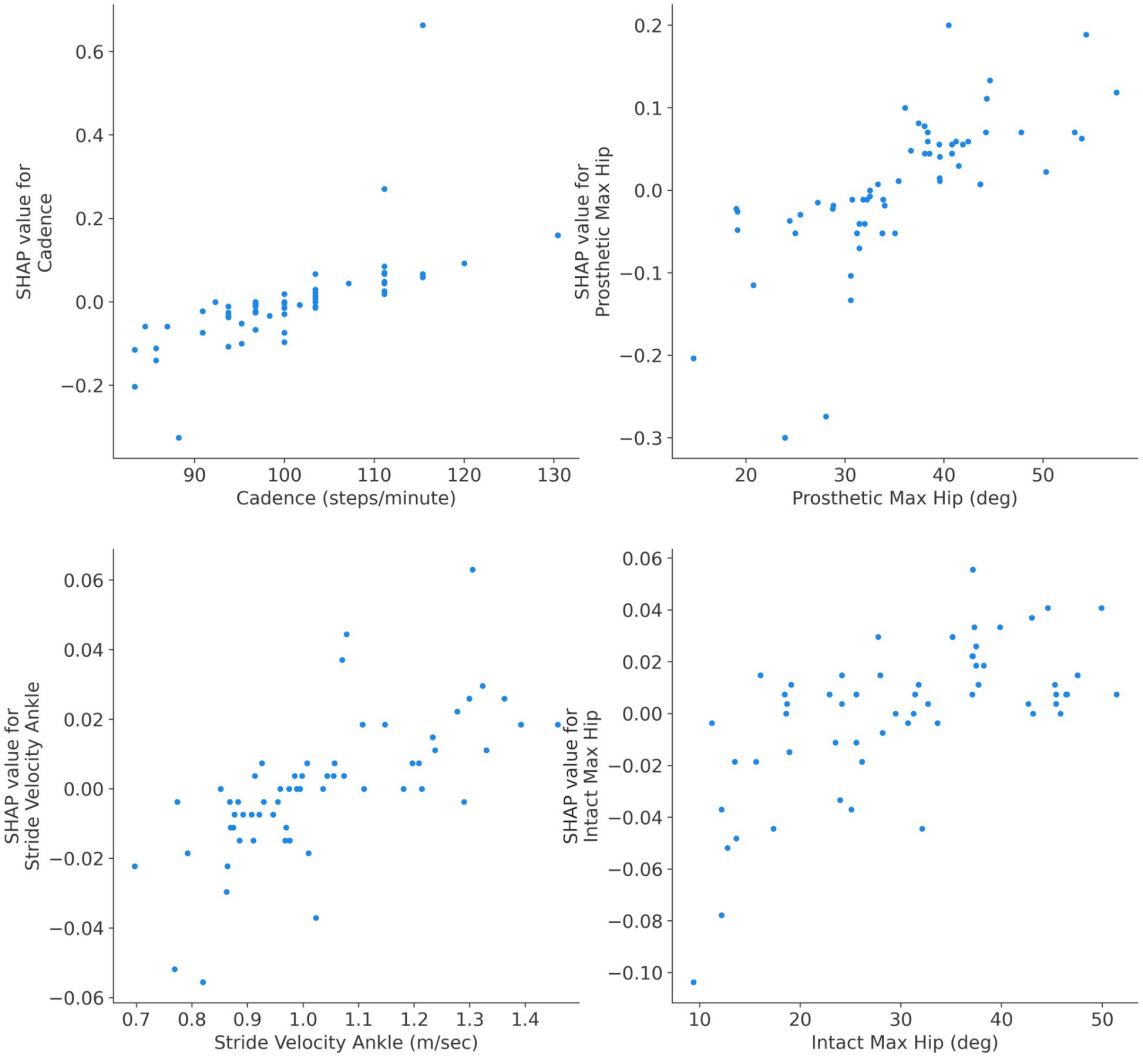
SHAP dependence plots for gait parameters ranked five to eight for the RF model. In general, all four gait parameters started from low values for ICRC knees and increased for the ATK knees. However, the trend and rate of the curves are different for different features. For example, stride velocity ankle shows a flat trend for the values between 0.9 m/sec and 1.1 m/sec.

**Fig 6:**
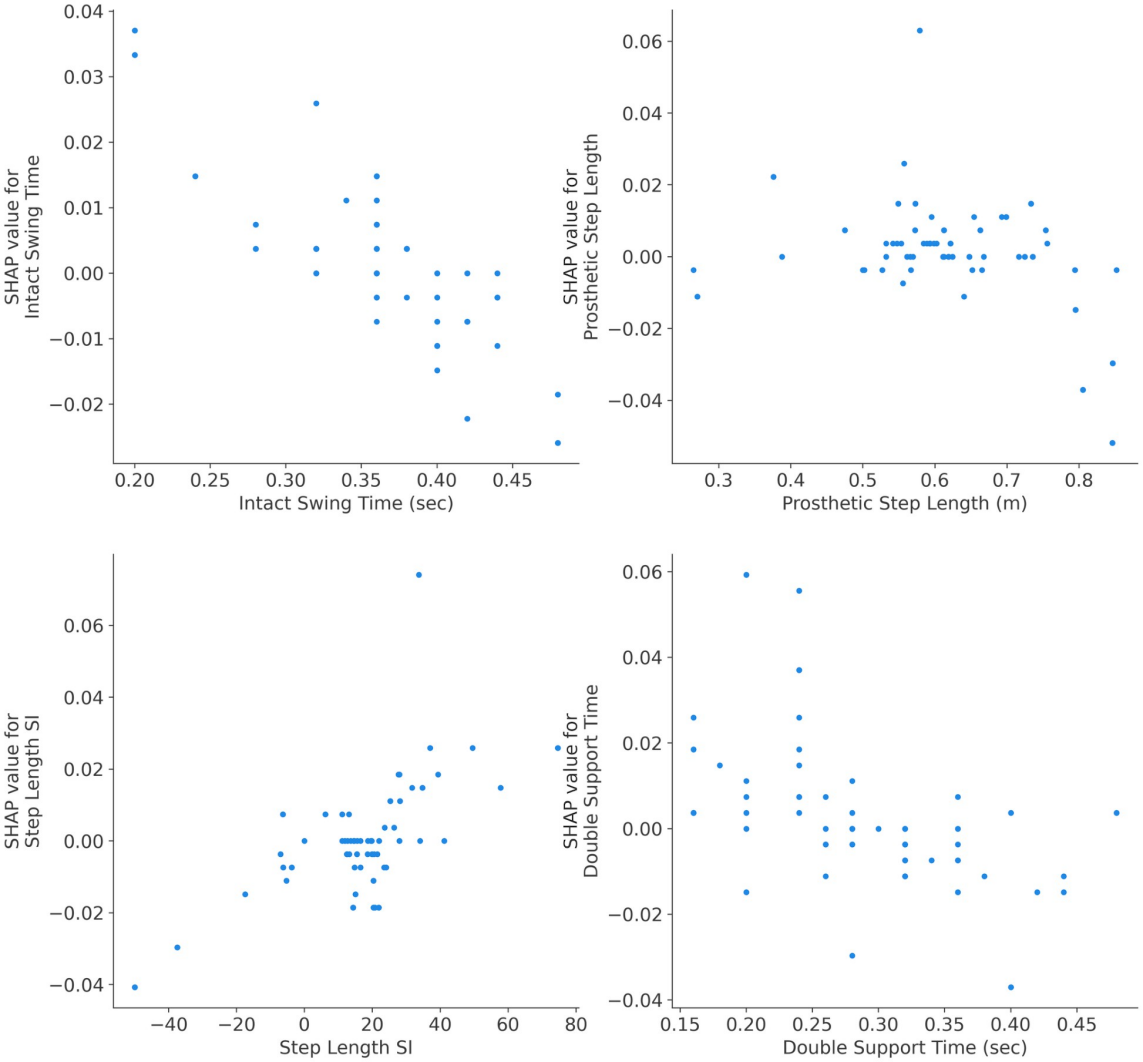
SHAP dependence plots for four least influential gait parameters via RF model. The general trend for the dependence plots of these features was flat indicating that these gait parameters had minimal impact on the model predictions. This means that neither ATK nor ICRC prosthetic knees affected these gait parameters. The dependence plot for prosthetic step length parameter demonstrates a nonlinear pattern between its varying values and the likelihood of wearing the prosthetic knees implying both ATK or ICRC knees could result in either low or high values of prosthetic step length (but the impact is low in any case).

According to the global interpretability results presented in Fig 3, SHAP dependences are plotted for the results of the best ML model (i.e. RF model). Fig 4 depicts dependence plots for four most influential gait parameters (four top features in Fig 3 for the RF model). In Fig 5, the dependence plots are illustrated for the second four most influential gait parameters (features ranked from five to eight in Fig 3 for the RF model). The dependence plots for four least influential gait parameters (last four features at the bottom of global interpretability plot in Fig 3 for the RF model) are in Fig 6.

### SHAP local interpretability: Explaining sample-based predictions

**Fig 7:**
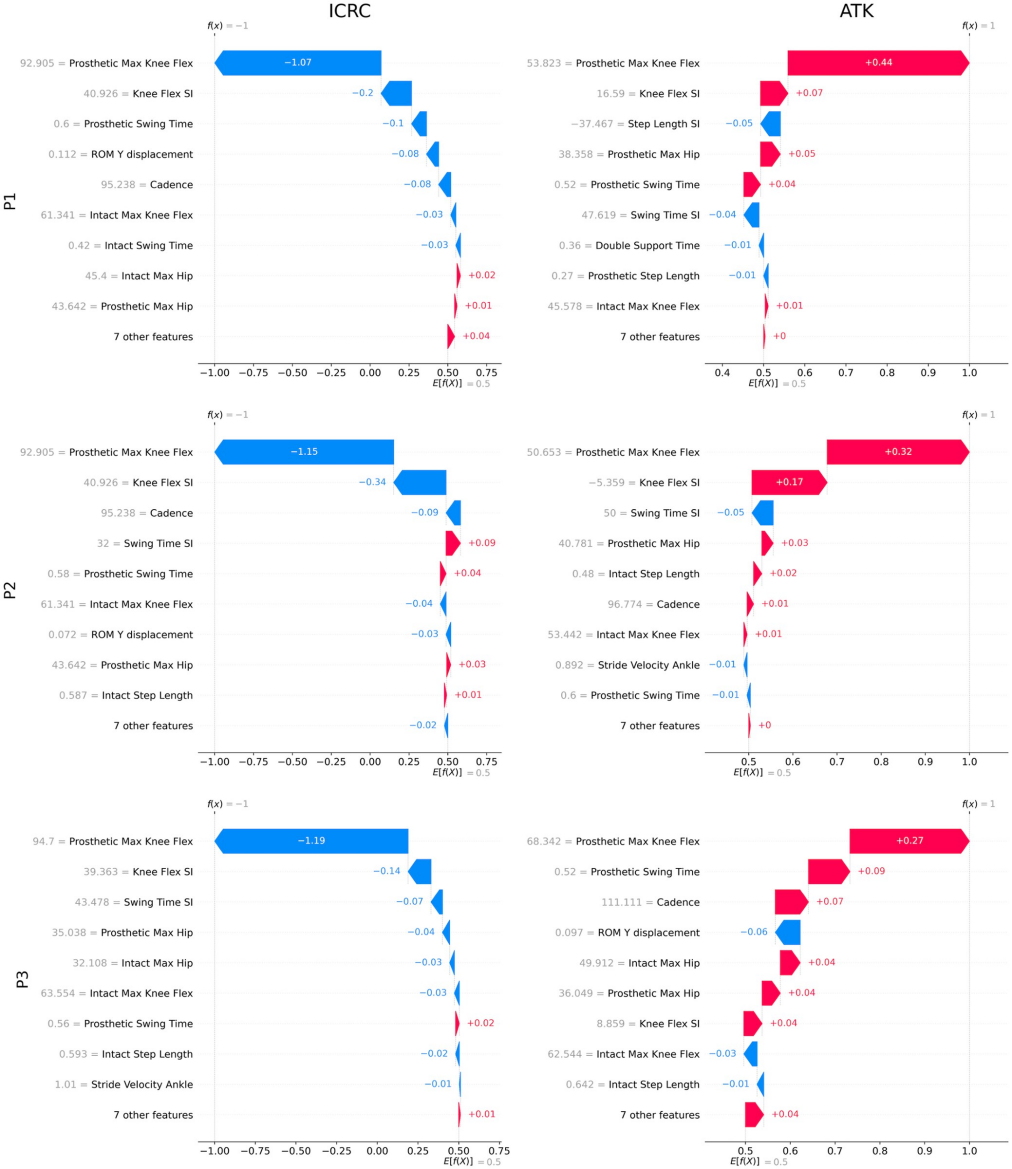
Local interpretability analysis for three samples using RF model. The red and blue arrows correspond to gait parameter values that push the model toward the ATK and ICRC knee class, respectively. The amount of variation of each gait parameter is determined by the length of the arrow (or the value associated with it). P1, P2, and P3 refer to three randomly selected study participants.

Local interpretability focuses on explaining classification outputs for sample participants. SHAP was used to explain how sample-based outputs (i.e. the label of each sample in the dataset) were reached through the contributions of each gait parameter. The results of local SHAP analysis obtained from the RF model on three different participants are illustrated in Fig 7. It was observed that in most cases the most contributing parameters were similar for both the ICRC and ATK knees but in the opposite direction. In other words, if a value of a gait parameter was reduced by a knee, its value is increased by the other knee with small discrepancies being attributed to randomness in the execution of the models. These mirrored results are expected in the simple example of two knee joints, while more sophisticated models (two or more interventions as may be relevant in many cases of prosthetic rehabilitation) could be expected to yield more complex relationships in the analyses.

Remark 4. In general, the predicted output of the developed models is a (semi)linear or nonlinear weighted aggregation of all the input features (i.e. gait parameters). Each model tries to minimize a loss function that is a function of total prediction errors. Since the target values are +1 and -1 for ATK and ICRC, respectively, models will focus on those input features that have high variations between two knees so that they can recognize (minimize the loss function or error) based on those features. Otherwise, a feature having similar value for both knees will not most probably be useful for the models as it will give similar prediction output for both knees and, therefore, the error will go high and the loss function will not be minimized. For example, for knee flex SI, which is one of the important features, there are only two possibilities, whereby one knee will be high and the other them must be low. If they were both similar, then inherently, the feature would not come out as being important at distinguishing the knee and, hence, the model loss function would not be minimized. Accordingly, the more/higher vs lower a parameter value is, the more important it is in the model. So, SHAP analysis will detect similar most contributing parameters for both ICRC and ATK knees in opposite directions to minimize the errors of the labels (as the labels are opposite each other). It is noted that the most contributing parameters could be different, if the labels of the knees were similar (i.e. if the models were trying to predict similar labels for the different knees).

**Remark 5**. The existence of red color arrows in ICRC knees in Fig 7 means that the corresponding gait parameter does not positively contribute toward the ICRC knee. Contribution means how much the corresponding gait parameter is influential on the prediction result (+1 or -1). For example, it is seen that the step length SI has been appeared with blue color for P1 in ATK class indicating that this parameter is recognized as an influential parameter for ICRC rather than ATK. This means that although the step length SI has the third place among the gait parameters, its contribution is leaned toward the ICRC knee. But since its effect is low (the size of the blue color arrow is small), the knee type is recognized as ATK. Also, a positive SHAP value (the number inside the arrows) indicates a feature’s positive contribution toward an ATK knee as it is labeled with +1, while a negative value indicates a positive contribution toward an ICRC knee as it is labeled with -1. In the meantime, red arrows direct to the right (ATK knees) and blue arrows aim at the left side targeting ICRC knees. A similar inference is valid for the blue color arrows in ATK plots (such as ROM Y displacement for P1 in Fig 7) in which the gait parameters with blue color arrows in ATK plots contribute toward the ICRC knees. But since their contributions are minor compared to the other gait parameters shown with red color arrows, the ultimate result of the global model is leaned toward the ATK knee.

**Fig 8:**
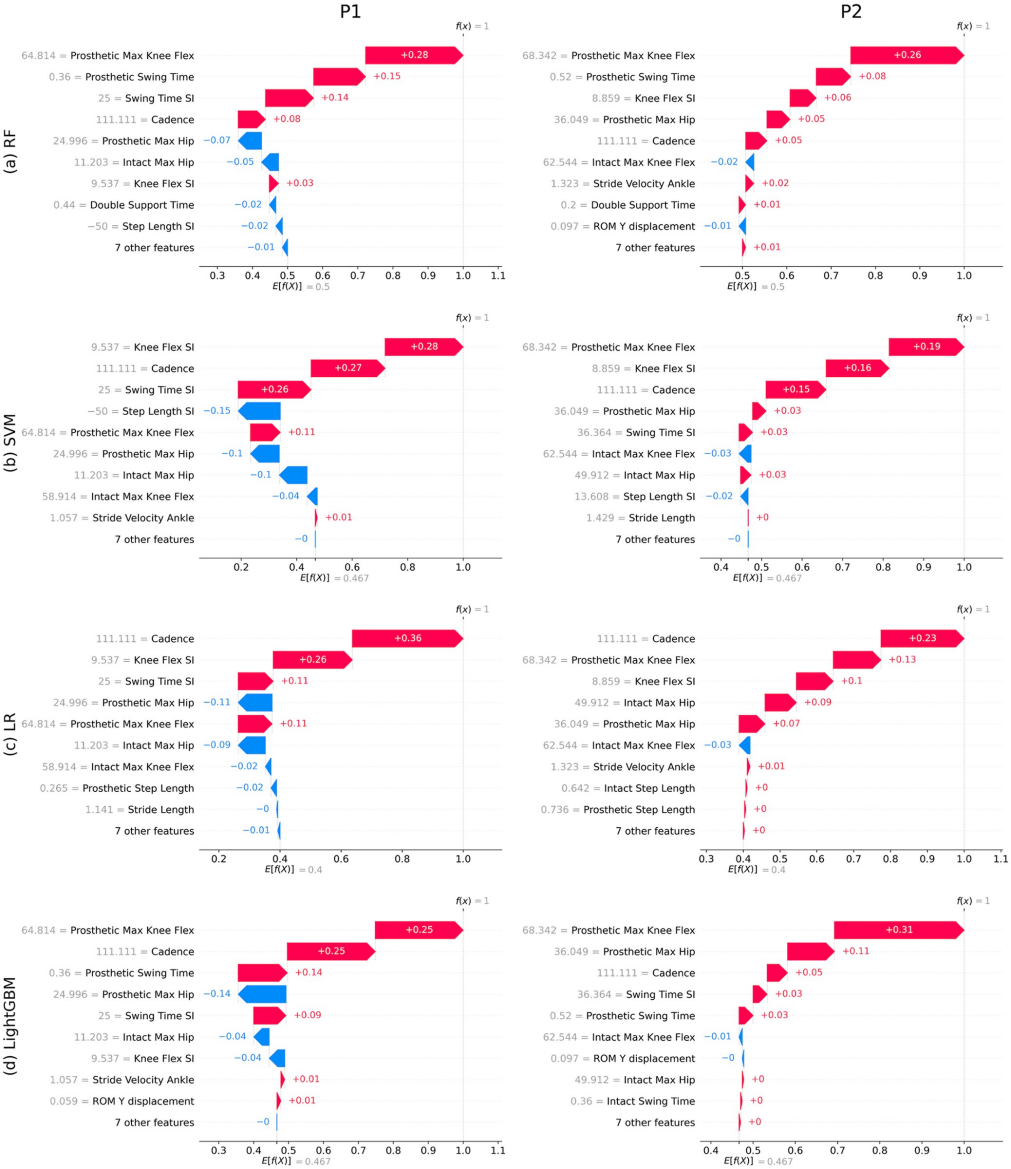
Local interpretability analysis for two samples wearing ATK prosthesis using the four models. P1 and P2 are randomly selected study participants.

Fig 8 shows the waterfall plots obtained from RF, SVM, LR, and LightGBM, respectively, for two different samples wearing ATK prosthesis. It could be seen that each sample had different most influential gait parameters associated with the prosthetic knee even if the same ML model was utilized. For instance, Fig 8(b) shows that, for a participant wearing the ATK, the knee flex SI was the most important parameter while the same model had obtained the prosthetic max knee flex as the main dominant gait parameter for another person. However, in Fig 8(c), the cadence was identified as the most influential gait parameter for both participants wearing the ATK prosthetic knee. This shows that regardless of the results of global SHAP analysis, each person could have had disparate dominant gait parameters with local SHAP analyses and different ML models.

**Remark 6**. In the plots of Fig 8 where the results were depicted for ATK knees, it is observed that the plots include a mixture of both red and blue color arrows. To elaborate on this pattern, some points should be taken into account as follows: (1) The length of an arrow (regardless of its color) shows the amount of the contribution of the corresponding gait parameter in the predicted class type. For example, the prosthetic max knee flex with the largest length is placed on the top of the plot in Fig 8(a) meaning that it is the most important parameter contributing in the model prediction; (2) For ATK knees, the red color arrows with larger lengths are placed in higher ranks, indicating that the corresponding gait parameters are most influenced by ATK knees; and (3) Although the knee types in Fig 8 were all recognized as ATK, some parameters that negatively contributed to the prediction of the ATK were shown by blue color arrows in Fig 8. However, the effects of these blue color parameters would be less compared to the parameters indicated by red color arrows. In conclusion, the shorter lengths of the blue color arrows in the plots for ATK knees in Fig 8 are indicative of their contributions in the model predictions as being minor and hence the model leans toward ATK knees (i.e. the model predicted +1 as the final result implying an ATK knee).

## Discussion

This study aimed to apply and demonstrate explainable ML-based models to gait analysis while identifying the most influential features (i.e. gait parameters). All models performed with an accuracy over 90%, with RF models performing slightly better. This could be due to RF models representing the data using nonlinear parallel decision trees, which are known to reduce both bias (through hyperparameter tuning) and variance [27]. This suggests that non-linear tree-based ML models may better deal with the data in comparison to linear/semi-linear models.

Building upon previous work, this study explored the feasibility of using ML models to determine the influence of interventions on input variables (gait parameters) in a prosthetic application. Through global SHAP analysis, it was determined that the parameters related to knee flexion (prosthetic max knee flex and knee flex SI) and its timing (prosthetic swing time and swing time SI), along with the walking speed (stride velocity ankle and cadence), were the features that most affected classification. Stride length, step length SI, and double support time were found to have minimal influence on the predictions of the classifiers. This suggests that the two knee types had similar outcomes for these gait parameters. These findings were consistent with those of [3], where the same two prosthetic knees were analyzed using traditional linear statistical methods. However, owing to the nonlinear nature of the adopted methodologies, the current work discovered certain nonlinear relationships between the gait parameters and the knee type. For example, it was shown that the ATK results in higher values of cadence, while the aforementioned study [3] obtained a marginal statistically significant effect (p-value = 0.02) for cadence. This might be due to an existing nonlinear relationship between cadence and the prosthetic interventions that could not be captured by linear methods.

Unlike traditional statistical analyses or black-box ML models, the presented explainable ML models were able to determine the amount of variation of each gait parameter affected by a specific prosthetic device using SHAP analysis. For example, the feature importance graphs (Fig 2) of tree-based methods determined the relative effects of different prosthetic devices on each gait parameter. Hence, a multiple-wise comparison between the affected gait parameters could be performed using SHAP analysis. Based on the importance values provided by SHAP analysis (e.g. the values associated with the arrows in Figs 7 and 8), the quantity of the various gait parameters could be ascertained for each intervention. Conventional statistical methods (e.g. ANOVA) do not generally provide the corresponding amounts of parameter variations caused by nonlinear interactions via different interventions. Therefore, the properties of SHAP analysis could complement the results of existing conventional statistical methods, specifically in cases with nonlinear and complex big data.

Incorporating SHAP dependence plots revealed the impact of a single gait parameter on the classification results of the models. From the analysis, it was observed that the trend in Fig 4 is decreasing, increasing in Fig 5 and staying flat in Fig 6. The direction of the trend (increasing or decreasing) shows the direction of the impact of the chosen feature on the model’s output. For example, the decreasing trend of knee flex SI in Fig 4 indicates that the large values of this gait parameter were associated with ATK knees and lower values belonged to the ICRC knees. On the other hand, the increasing trend of prosthetic max hip in Fig 5 revealed that ATK knees decreased the amount of maximum hip compared to the ICRC prosthesis. Also, the flat trend in Fig 6 suggested little to knee type had no significant impact on the corresponding gait parameter. The nonlinear relationship between a gait parameter and the type of a prosthetic knee could be identified via SHAP dependence plots too. For instance, Fig 4 suggests a nonlinear relationship between swing time SI and the adopted prosthetic knee. A (semi)linear relationship between stride velocity ankle is seen in Fig 5. Reveal of both (semi)linear and nonlinear relationships between gait parameters and the knee types by SHAP dependence plots suggests the use of a nonlinear model for classifying the knees.

**Fig 9:**
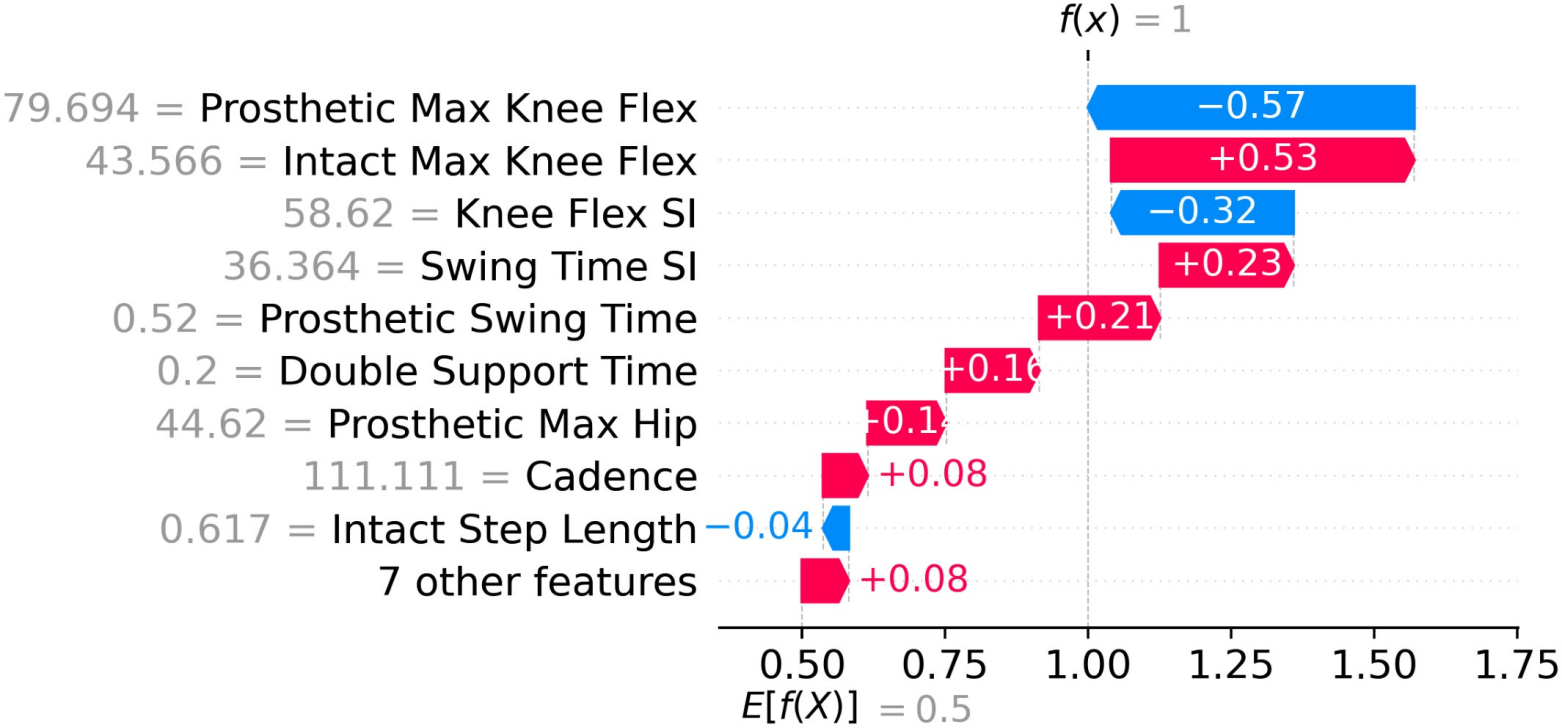
SHAP analysis for a false positive example of classification for a single participant wearing an ICRC prosthesis obtained from the SVM model. Since the label -1 was assigned to the ICRC, one expected to see a vertical line labelled with -1. However, the class label was marked +1, meaning that the model had identified this sample with an ATK prosthesis.

The local interpretability (sample-based) analysis of the explainable ML methods presented in this study provides powerful insight into the effect of knee type on the walking style of a specific sample, whether or not it is in agreement with global (population) interpretations. An example of this incongruency was seen in the rank of the cadence in Figs 3(b) and 8(b). It could be observed that the rank of the cadence was changed compared to the global interpretability results in which its place was upgraded from third in global analysis to first in local SHAP plots for the SVM model. This reveals the importance of sample-based interpretability analysis, as the local results did not align with the global results for the given sample. This could be useful for analyzing and developing rehabilitation processes for a specific sample (i.e. personalized health care). Moreover, local explanations could also be beneficial in critical cases, where the analysis of waterfall plots can identify false positive or negative outcomes as misclassified and misleading situations [30]. For instance, the local SHAP analysis for a participant wearing an ICRC prosthetic obtained from the SVM model was illustrated in Fig 9. This figure shows prosthetic max knee flex with a blue color arrow as the most important gait parameter affected by the worn prosthetic and determined the prosthesis type as ATK (the label on the vertical line was +1). However, although the most effective gait parameter for the identified prosthesis type (i.e. ATK prosthesis for the SVM model in Fig 3) was prosthetic max knee flex, the color of arrow should have been red indicating that high values of this parameter were associated with ATK knees. The same discrepancy could be observed for the knee flex SI where it was associated for an ATK knee with a blue color arrow in Fig 9, while the global plot in Fig 3 determined that blue color for this gait parameter should be associated with ICRC knees. Thus, it could be concluded that this particular prediction was a false positive case where the model misclassified the ICRC prosthesis as an ATK knee and, therefore, incorrect importance results were found through SHAP. In such circumstances, and to capture the meaningful sample or sub-group differences, more accurate classifiers—such as deep learning methods (if sufficient data is available) or a combination of models—can be used to improve the reliability of the results.

This study had certain limitations. First, the accurate capture and calculation of certain gait parameters were difficult to achieve using the implemented video-based gait analysis method [31]. Some minor discrepancies existed in the obtained interpretability results. For example, the ranks of less important gait parameters in the SHAP plots in Fig 3 (i.e. the parameters located in the lower places in the plot) were different for various models. In general, authentic characteristics of the interpretability experiments (i.e. SHAP analysis) can be established if different methodologies result in similar conclusions in terms of both accuracy and the interpretability. On the other hand, the results of the SHAP analysis might differ for various runs of the same ML classifier due to either the existing randomness in the procedure of the ML algorithms (e.g. weight initialization) or different hyperparameters chosen for the model. In order to handle the randomness of the implementations, random seed was set to a unique value to reproduce the same results in different runs. In cases where setting seed was insufficient for obtaining reproducible results, multiple runs with averaged results were used. Also, the ML model that obtained the best accuracy for the intervention classification was be selected as the final decisive model, and the corresponding feature importance results could be considered as the indicators of the significance of the gait parameters. Additionally, the parameters related to Hip (including Max/ROM Hip) were removed from the dataset to examine the accuracy and interpretability of the revised models. However, it was found that the tuning of the hyperparameters of the models yielded similar results. Thus, since these parameters were dropped from the dataset, the effects of the different interventions on them could not be verified. Hence, as part of future work, the inclusion and exclusion of other gait parameters, as well as sensitivity and robustness analysis, should be conducted.

## Conclusion

Explainable ML techniques can enable researchers and clinicians to better understand the effects of interventions on important patient outcomes, such as gait quality, as presented in this study. This study that both global and local interpretability analyses can be useful, providing generalizable information relating to a certain population, and also facilitating the assessment of sample patients who may present differently from the population. This research has demonstrated the potential use of explainable ML models in the assessment of clinical interventions, particularly the influence of a prosthetic knee joint on gait, which can be extended to and utilized in other clinical and non-clinical applications.

## Supporting information

S1 File Inclusivity in global research. (DOCX)
